# An Analysis of Hydromorphological Index for Rivers (HIR) Model Components, Based on a Hydromorphological Assessment of Watercourses in the Central European Plain

**DOI:** 10.1007/s00267-022-01778-6

**Published:** 2023-01-17

**Authors:** Tomasz Garbowski, Adam Brysiewicz, Justyna Nosek, Dominika Bar-Michalczyk, Przemysław Czerniejewski

**Affiliations:** 1 Institute of Technology and Life Sciences – National Research Institute Falenty, 3 Hrabska Avenue, Raszyn, 05-090 Poland; 2 Mineral and Energy Economy Research Institute, Polish Academy of Sciences, Wybickiego 7A Street, Cracow, 31-261 Poland; 3 West Pomeranian University of Technology in Szczecin, Department of Commodity, Quality Assessment, Process Engineering and Human Nutrition, 4 Kazimierza Królewicza Street, Szczecin, 71-550 Poland

**Keywords:** Hydromorphological assessment, Poland, Water Framework Directive, Lowland river hydromorphology, Hydromorphological diversity, River ecosystem

## Abstract

Assessing the hydromorphological conditions of watercourses is a requirement of the Water Framework Directive (WFD) and national river status monitors (e.g., in Poland,the State Environmental Monitoring, and Water Monitoring coordinated by Chief Inspectorate of Environmental Protection). This paper evaluates the hydromorphological status of 10 watercourses (30 measurement sections) in Poland based on the multimetric Hydromorphological Index for Rivers (HIR). A new approach to the delineation of the river valley (small watercourses) is proposed. An analysis of the influence of river valley management on the value of HIR and its components was carried out using statistical methods (basic statistics, Mann–Whitney U Test and Ward’s cluster analysis). In addition, the relationship between the components of the HDS (Hydromorphological Diversity Score) and HMS (Hydromorphological Modification Score) was analyzed (Spearman’s Rank Correlation Coefficient). HIR values for the watercourse sections ranged from 0.553 to 0.825. HDS values ranged from 27.5 to 75.5 and HMS from 2.0 to 17.5. The results of the basic statistical analyses showed slight differences between the two river valley delineation methods. The Mann–Whitney U Test showed a significant difference in the test significance level of the HDS, HMS and HIR for the river valley delineation methods. Spearman’s rank correlation analysis showed that most of the HDS and HMS parameters components had a low degree of correlation. The juxtaposition of the two methods for delineating a river valley and its influence on the HIR allows for a better understanding of the interdependence between its parameters.

## Introduction

River ecosystems are centers of biodiversity providing habitats for the aquatic communities and ecosystems native to them, but are heavily influenced by anthropopressure due to agricultural intensification, water pollution and hydrotechnical structures (Gostner et al. 2013; Teufl et al. 2013; Belletti et al. 2018; Benadda et al. 2022), flood protection and others. Worldwide, these crucial sites have been significantly altered and biodegraded in recent decades due to the expansion of hydroelectric facilities and small hydropower plants, bottom dredging, and the transformation of riverbanks and riverbeds (Best 2019; Štefunková et al. 2020; Müller et al. 2022). Diverse watercourse restoration projects are being introduced to improve the quality and quantity of habitat and spawning sites for fish (Alokhina 2020; Nazari Giglou 2021). To implement conservation measures and set goals for surface water restoration, the river’s hydromorphological condition is assessed (Zaharia et al. 2018; Munoth and Goyal 2020; Lemay et al. 2021). This is a complex work algorithm that includes a detailed analysis of available source materials, the delineation of study sections, field surveys, and a final assessment of hydromorphological conditions (Raven et al. 1998). Various methods developed independently in different countries are used for this purpose, including the RHS (River Habitat Survey) in the UK (Environment Agency 2003), the LAWA (Habitat Assessment for Rivers) in Germany (LAWA 2002), the QBR (the Index of Riparian Quality) in Spain (Munné et al. 2003; Garcia-Burgos et al. 2015), the HEM (Hydroecological Monitoring Method)in the Czech Republic (Langhammer 2014; Kujanová et al. 2016), and the MQI (The Morphological Quality Index) in Italy (Rinaldi et al. 2016). In Poland, the HIR (Hydromorphological Index for Rivers) method was devised in 2017 to monitor the quality of watercourses (Szoszkiewicz et al. 2016, 2017, 2020). The HIR model meets the requirements of the Water Framework Directive (WFD) and European and Polish standards for the hydromorphological characteristics of rivers (EN 14614:2004 2004; PN-EN-14614:2008 2008; The Water Law Act of 2017; Decree of Minister of Infrastructure of 2021).

The main use of the HIR model is to monitor hydromorphological water quality characteristics. HIR can also be used to assess anthropopressure and the effectiveness of watercourse restoration methods. The Hydromorphological Index for Rivers (HIR) can be calculated for all types of flowing waters allowing the assessment of both low-altitude rivers, mid-altitude rivers, and mountain high-altitude streams. It can be used to assess natural as well as heavily altered watercourses and artificial channels (Szoszkiewicz et al. 2017, 2020). An additional use of HIR is to study ecological conditions for aquatic organisms (Przesmycki et al. 2017; Szoszkiewicz et al. 2017, 2020). The features of the HIR model proved its versatility, due to the wide spectrum of criteria and hydromorphological conditions considered during the assessment. The HIR can be used to assess the hydromorphological conditions of watercourses located in different parts of Europe (Tomczyk et al. 2021).

Many components of the HIR model are derived from the RHS model designed in the United Kingdom (Szoszkiewicz et al. 2020). However, the HIR has some advantages over the baseline method. The RHS method is based only on field observations of short survey sections (Raven et al. 1998; Davenport et al. 2004; Wiatkowski and Tomczyk 2018) which often fail to fully represent the current condition of the watercourses (Osowska and Kalisz 2011). The HIR model also uses GIS (Geographic Information System) data (included in Szoszkiewicz et al. 2020) so that it can provide more detailed results. In developing the HDS and HMS indices, an attempt was made to eliminate the shortcomings of their RHS counterparts by, for example, expanding the list of scored seminatural land uses and differentiating the scoring of hydraulic structures depending on the degree of environmental impact (Environment Agency 2003; Szoszkiewicz et al. 2017). In addition, compared to the RHS, the precision of the HIR model was increased by differentiating hydromorphological unit types and cross-section of the riverbed, and by adding a parameter: the width of the unused coastal zone (Szoszkiewicz et al. 2016).

This paper evaluates the hydromorphological condition using the HIR model and statistically analyzes the relationship between the model components. The analysis focused on the mode and nature of river valley use, which influences river morphological conditions. Authors of previous studies have analyzed the effect of river valley use on the HIR index (Pietruczuk et al. 2019, 2020). They determined the river valley buffer according to the HIR methodology guidelines (Szoszkiewicz et al. 2017). This paper examines whether the change in the definition of the “river valley” (Szoszkiewicz et al. 2016, 2017, 2020) will affect the assessment of the hydromorphological condition of the river. The statistical analyses were made using two methodologies for river valley delineation.

## Materials and Methods

### Characteristics of the Study Area

The study area was in the central and eastern part of the Central European Plain, in the Southern Baltic Lake District/Costal Region and the Masovian Plain. The study was conducted on ten watercourses located in the Odra River and Vistula River basins. Detailed characteristics of the study sections are given in Table 1.

**Table 1:** Characteristics of the watercourses in the designated sections used in the HIR model

Measurement point	Flow rate [cm s^−1^]	Flow [cm^3^ s^−1^]	Watercourse length [km]	Width of riverbed [m]	Bottom depth [m]		Water table drop [‰]	Catchment area [km^2^]	Land use and characteristics of catchment
					in relation to the left/right bank	in relation to natural land left/right			
Myśla −1	0.359	0.091	95.6	1.57	1.25/1.25	1.95/2.45	2.0	154.81	A—68%, FO—22%, M—7%, U—1%, W—1%, MA—1%
Myśla −2	0.310	0.070		1.93	0.85/1.10	2.00/1.90	3.0	143.16	
Myśla −3	0.184	0.038		1.05	3.90/4.30	3.90/4.10	3.0	111.07	
Płonia −1	0.699	1.024	72.6	4.33	2.90/2.60	3.55/3.45	5.0	367.04	A—54%, FO—30%, M—8%, U—5%, W—2%, MA—1%
Płonia −2	0.194	0.930		8.34	2.30/2.15	2.30/2.45	3.7	174.59	
Płonia −3	0.185	0.781		6.30	2.65/2.55	1.65/1.70	2.0	143.59	
Rurzyca −1	0.209	0.242	44.4	4.13	1.65/1.35	1.65/1.45	3.0	87.45	A—58%, FO—24%, M—12%, U—3%, MA—2%, W—1%
Rurzyca −2	0.321	0.433		4.00	1.80/1.60	1.55/1.50	3.0	83.03	
Rurzyca −3	0.395	0.330		2.57	1.90/2.00	2.00/2.25	1.0	68.41	
Tywa −1	0.419	1.142	48.5	5.33	1.25/1.50	1.85/2.40	0.2	274.25	A—57%, FO—28%, M—7%, U—4%, W—3%, MA—1%
Tywa −2	0.705	0.866		4.30	1.10/1.30	2.45/1.90	2.4	270.23	
Tywa −3	0.132	0.058		1.63	1.60/1.50	2.10/2.15	0.8	16.63	
Wardynka −1	0.430	0.062	20.3	1.97	1.20/0.95	0.45/1.00	1.0	25.93	FO—51%, A—36%, M—13%
Wardynka −2	0.696	0.143		2.90	0.50/0.75	1.45/2.05	6.8	25.16	
Wardynka −3	0.232	0.015		1.13	1.75/1.20	1.85/1.65	0.5	3.27	
Czarna-Cedron −1	0.130	0.110	14.5	3.30	2.25/2.15	3.05/2.35	0.5	73.80	O—34%, FO—32%, A—18%, U—13%, M—3%
Czarna-Cedron −2	0.089	0.271		5.07	1.35/1.00	1.95/1.00	0.5	69.48	
Czarna-Cedron −3	0.069	0.215		5.67	2.55/2.45	2.90/2.25	0.5	68.41	
Kanał Habdziński −1	0.183	0.803	7.0	7.57	2.40/2.35	2.45/2.20	0.5	28.08	A—56%, M—16%, U—16%, FO—7%, MA—5%
Kanał Habdziński −2	0.184	0.531		6.03	1.60/3.00	2.90/2.65	0.5	21.57	
Kanał Habdziński −3	0.027	0.018		2.90	1.10/3.20	1.65/3.25	1.8	11.03	
Kraska −1	0.192	0.100	28.8	2.23	2.00/1.90	2.00/1.75	3.5	27.50	O—44%, A—30%, FO—14%, M—8%, U—4%
Kraska −2	0.148	0.030		1.57	1.35/1.65	1.35/1.55	3.8	27.14	
Kraska −3	0.122	0.068		2.43	0.95/1.20	1.00/1.25	3.7	24.81	
Molnica −1	0.125	0.006	14.6	0.67	1.60/0.90	1.80/1.10	3.0	13.79	O—68%, A—19%, FO—13%
Molnica −2	0.056	0.001		1.00	0.85/1.20	1.15/1.05	3.0	13.25	
Molnica −3	0.143	0.012		1.23	1.05/0.85	1.00/1.05	5.8	8.46	
Zielona −1	0.118	0.026	11.9	2.60	1.75/1.25	1.45/1.45	1.7	38.21	A—59%, M—19%, FO—13%, U—7%, O—2%
Zielona −2	0.179	0.026		2.40	1.20/1.10	1.05/1.20	0.8	26.72	
Zielona −3	0.248	0.061		1.73	1.45/1.15	1.30/1.20	0.8	19.37	

*A* arable land, *M* meadows, *O* orchards, *FO* forest, *U* urban areas, *W* water (reservoirs, rivers), *MA* marshes

**Fig. 1:**
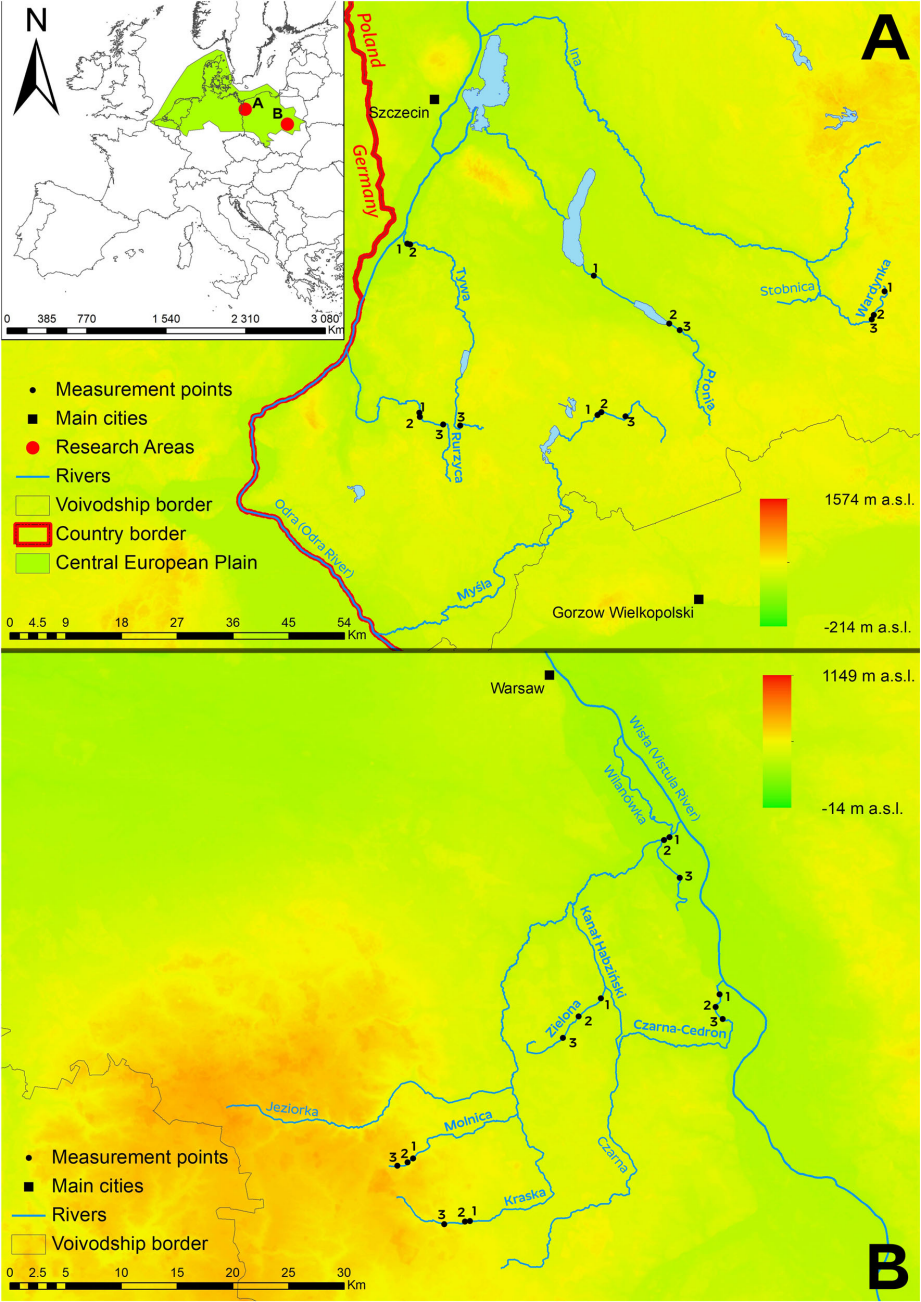
Rivers in the Central European Plain for HIR analysis: **A** – rivers in the Odra River catchment, **B** – rivers in the Vistula River catchment

Of the rivers studied, 5 were located in the Southern Baltic Lake District/Costal Region in the Lower Odra and Western Pomerania water region: Myśla, Płonia, Rurzyca, Tywa, Wardynka (Fig. 1A) and 5 in the Masovian Plain in the Middle Vistula water region: Czarna-Cedron, Kanał Habdziński, Kraska, Molnica, Zielona (Fig. 1B). Three representative 500 m sites were designated on each river (Brysiewicz et al. 2018, 2019) where analyses were conducted during the summer (August) of 2017. The longest of the rivers is the Myśla River (95.6 km), with the shortest the Kanał Habdziński (7.0 km). The watercourse sections were selected on the basis of their physiographic features and the land use type of valley. All the selected sections are located in lowland areas. The channel width for each of the watercourses does not exceed 30 m. The common physiographic features made it possible to compare the results of the analysis using the HIR model. The varying proportions in the type of land use in the catchment will make it possible to compare its influence on the hydromorphological assessment index of the watercourses.

It is worth mentioning that studies using the RHS model have previously been conducted on these watercourses (Brysiewicz et al. 2019) and the data collected formed the basis for the hydromorphological assessment using the HIR model.

### HIR Model Methodology

The HIR analysis is based on hydromorphological information (Table 12) from selected watercourse sections collected during the field survey. This information needs to be supplemented with data from source materials (orthophoto maps, GIS data, reports on the state of the Surface Water Bodies) that allow for an assessment of the whole Surface Water Bodies (SWB). The HIR index is multimetric, based on two sub-indices (HDS and HMS). The Hydromorphological Diversity Score (HDS) reports the heterogeneity of the river ecosystem and considers 13 parameters related to the riverbed zone, riparian zone and river valley. The Hydromorphological Modification Score (HMS) indicates the degree of naturalness in the river ecosystem and has 5 parameters: a transformed cross-section of the riverbed, hydrotechnical structures, transformations in control profiles, impediments to the connectivity of the river with the valley and other anthropogenic pressures. A GIS data assessment can be performed without having to carry out a field assessment. In order to make a hydromorphological assessment of the entire SWB from a GIS data assessment, publicly available spatially oriented databases (GIS) from national surface water monitoring or SWB condition monitoring are used (Hydroportal ISOK 2015; Szoszkiewicz et al. 2017; Pietruczuk et al. 2020; MPHP 2021; BDOT10k 2022; Geoportal GUGiK 2022; Geoportal NWMA 2022; Geoservice GDEP 2022).

The results of the field assessment are two separate indices, the HDS_f_ and HMS_f_. Both indices are components of the HIR multimetric, which is the result of the field assessment. It is calculated from the formula (Przesmycki et al. 2017; Szoszkiewicz et al. 2017, 2020):

$$HIR = \frac{{\left( {{\textstyle{{HDS_f - HMS_f} \over {100}}}} \right) + 0.85}}{{1.8}}$$

where:

HIR - Hydromorphological Index for Rivers

HDS_f_ - Hydromorphological Diversity Score based on field assessment

HMS_f_ - Hydromorphological Modification Score based on field assessment

The value of HIR is in the range of 0–1, where 0 indicates extreme hydromorphological transformation and 1 is the reference value. The HIR multimetric thus calculated allows the river to be classified into one of five hydromorphological status classes based on the hydromorphological type of the river (Table 2).

**Table 2:**
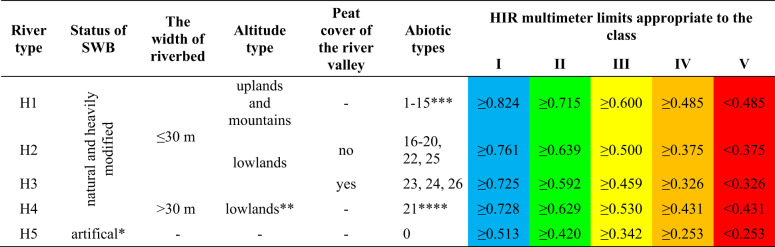
HIR multimetric breakpoints for five hydromorphological state classes (Szoszkiewicz et al. 2017, 2020)

* - it does not include artificial dam reservoirs for which the river SWB was created

** - also includes 5 SWBs with a riverbed width >30 m, located on the San River (abiotic types 14 and 15)

*** - excluding 5 SWBs with a riverbed width >30 m, located on the San River (abiotic types 14 and 15)

**** - also includes other SWBs with a riverbed width >30 m, representing abiotic types: 14, 15, 19, 24 and located on the rivers: Wisła, Odra, Warta, Narew, San, Noteć and Wieprz

The result of the GIS data assessment, like the field assessment, is the Hydromorphological Diversity Score (HDS_GIS_) and the Hydromorphological Modification Score (HMS_GIS_) within which the Hydromorphological Diversity Score Attributes (HDSA) and the Hydromorphological Modification Score Attributes(HMSA) are defined. The final GIS data assessment is made by calculating a Correction Factor (C_f_) based on the Hydromorphological Diversity Score (HDS_GIS_) and the Hydromorphological Modification Score (HMS_GIS_). The individual indices were calculated using the formulae (Szoszkiewicz et al. 2017, 2020):

$$HDS_{GIS} = {\sum} {HDSA_i}$$

where:

HDS_GIS_ - Hydromorphological Diversity Score based on GIS data assessment

HDSA - Hydromorphological Diversity Score Attributes

i - consecutive HDSA, from 1 to 7

$$HMS_{GIS} = {\sum} {HMSA_i}$$

where:

HMS_GIS_ - Hydromorphological Modification Score based on GIS data assessment

HMSA - Hydromorphological Modification Score Attributes

i - consecutive HMSA, from 1 to 6

$$Cf = \frac{{\left( {{\textstyle{{HDS_{GIS} - HMS_{GIS}} \over {10}}}} \right) + 1,2}}{3}$$

where:

C_f_ - Correction Factor for hydromorphological state class based on GIS data assessment

HDS_GIS_ - Hydromorphological Diversity Score based on GIS data assessment

HMS_GIS_ - Hydromorphological Modification Score based on GIS data assessment

C_f_ values range from 0 (extreme hydromorphological transformation) to 1 (reference value). Depending on the value of the Correction Factor (C_f_), the class resulting from the multimetric HIR calculated during the field assessment can be increased or decreased by 1 (Table 3) (Szoszkiewicz et al. 2017; Pietruczuk et al. 2019; Szoszkiewicz et al. 2020; Tomczyk et al. 2021; Borek and Kowalik 2022).

**Table 3:**
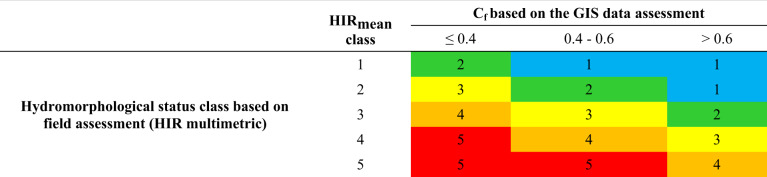
Changes in the HIR multimetric class depending on Correction Factor value (Szoszkiewicz et al. 2017, 2020)

### Statistical Analysis of Watercourse Hydromorphological Assessment Results

Statistical analysis was performed using the HDS, HMS and HIR indices calculated from the field form. STATISTICA 14 software was used for the statistical analyses. These included the calculation of minimum and maximum, quartiles, median and dominant values for the watercourse hydromorphological assessment indices. In addition, Spearman’s rank correlation analysis was performed for the components of the HDS and HMS. Spearman’s equation for tied ranks was used for analysis:

$$r_S = 1 - \frac{{6\mathop {\sum}\nolimits_{i = 1}^n {d_i^2 + T_x + T_y} }}{{n\left( {n^2 - 1} \right)}}$$

where:

r_S_ - Spearman’s Rank Correlation Coefficient between variables X and Y

d_i_ - rank difference between the same observations for two variables

n - number of observations

$$T_x = \frac{1}{{12}}\mathop {\sum}\limits_i {\left( {t_i^3 - t_i} \right)}$$

$$T_y = \frac{1}{{12}}\mathop {\sum}\limits_i {\left( {u_i^3 - u_i} \right)}$$

where:

t - number of observations with the same rank for X variable

u - number of observations with the same rank for Y variable

### Delineation of River Valley Zones Using Two Different Methods

**Fig. 2:**
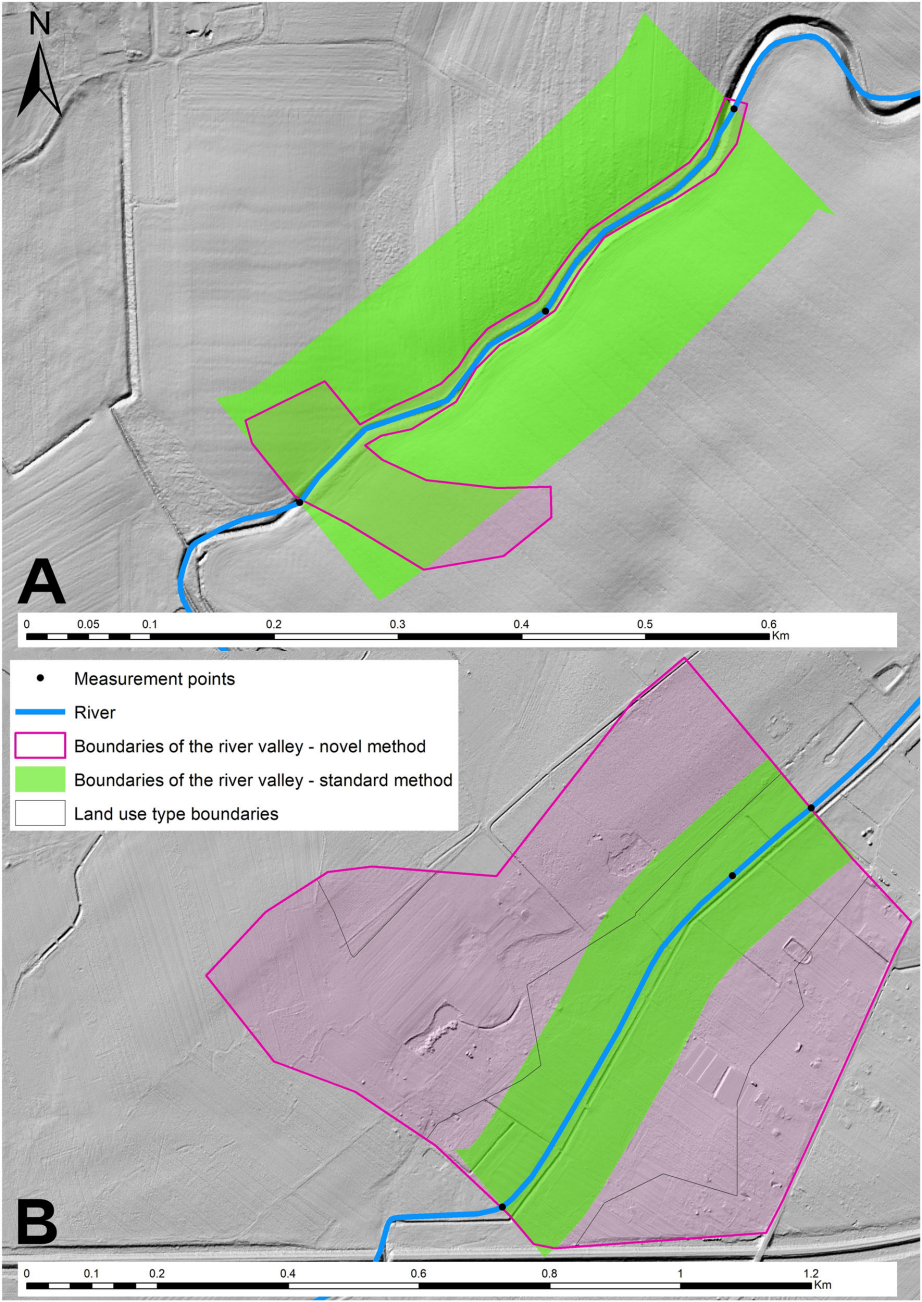
Example of change to the river valley area depending on the way it was delineated – extreme cases – (**A**) area of the river valley delineated using the standard method > area of the river valley delineated using the novel method (Myśla River) (**B**) river valley delineated using the standard method < river valley delineated using the novel method (Zielona River)

Delineation of the river valleys to assess their management type was carried out using two different methods. The first is based on the guidelines in the HIR manual (Szoszkiewicz et al. 2017). It assumes a 100 m wide buffer along a river with a riverbed width ≤30 m. Then, in this zone, the proportion of urbanized (U), agricultural (A) and seminatural (S) land is assessed and the dominant land use type is determined (if its proportion in the total area is >25%). The advantage of this method is its repeatability, but it is based on a large approximation. The standard method is based on a simple geoprocessing operation in GIS software. This operation results in a polygon of relative equal width on both sides of the watercourse (Fig. 2 – green polygon). For highly regulated rivers, the area determined by this method may be close to reality. However, in other cases, the use of this approach may lead to the designation of a completely different area of the valley than the real one, and thus different proportions between land use types in this zone. Therefore, a second method (novel method) of determining the river valley has been proposed.

The dominant land use type: urbanized (U), agricultural (A), seminatural (S) is determined in the same way for both standard and novel methods. The difference between the standard and novel method lies in the way the river valley is delineated (Fig. 2 – purple polygon). In the novel method the river valley is also delineated using GIS software, only instead of using the geoprocessing operations, a detailed analysis of topography and land cover is made. The data used to determine it were the Polish Hydrographic Map at a scale of 1:50,000, topographic maps at a scale of 1:10,000 and high-resolution orthophoto maps. The main intention during the delineation process was to define the zone of interaction between ground and surface waters where direct runoff of the surface and subsurface to the watercourse occurs. The process of delineating a river valley was done manually. Attention was paid to the nature of the riverbed and its geometry (regulated, flat, (wide, narrow) and topographic limitations of the river valley (road embankments, slopes, impermeable watercourse bed), which may limit the zone of direct runoff of the surface and groundwater to the watercourse. The valley boundaries were based on such barriers or on topography. As a result, for each section of the watercourse, a polygon covering its direct valley was established. In contrast to polygons designated by the standard method, polygons from the novel method had irregular shape and varied distance from the border of the area to the watercourse bed. Depending on the knowledge and experience of the researcher, even when using the same data the valley determined in this way may have a partially different course and different area.

The Mann–Whitney U Test for different types of river valley management and for different methods of delineating a river valley was done. Moreover, cluster analysis (Ward’s method) was made for each section studied (Pietruczuk et al. 2020). Urbanized areas were not included in the Mann–Whitney U Test because they were only present at one study point.

## Results

### HIR Values for the Watercourse Sections based on the Field Assessment

The rivers in the Masovian Plain showed smaller variations in hydromorphological status compared to those located in the Southern Baltic Lake District/Costal Region (Tables 4 and 5). Most of the sections studied had good hydromorphological diversity. Tables 4 and 5 summarize the multimetric HIR values, which range from 0.553 (Molnica-2) to 0.825 (Wardynka-2). These rivers are in two different regions of Poland. The rivers in the Southern Baltic Lake District/Costal Region were characterized by better hydromorphological status than those in the Masovian Plain. Additionally, the Hydromorphological Diversity Score (HDS) and Hydromorphological Modification Score (HMS) values are provided in Tables 4 and 5. The highest HDS value was found for the Wardynka-2 at 75.5, with the lowest value for the Kraka-2 and the Tywa-3 at 27.5, which indicates a higher degree of hydromorphological degradation in these sections compared to the Wardynka-2. The HMS was highest for the Molnica-2 (HMS = 17.5). The lowest value was recorded for the Tywa-2 (HMS = 2.0), which means that in this section morphological changes are small.

**Table 4:**
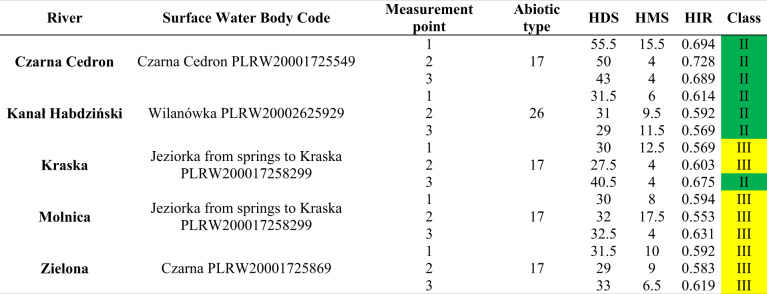
Results of the field assessment for the rivers studied in the Masovian Plain. The color of the class values corresponds to the color from Table 2

**Table 5:**
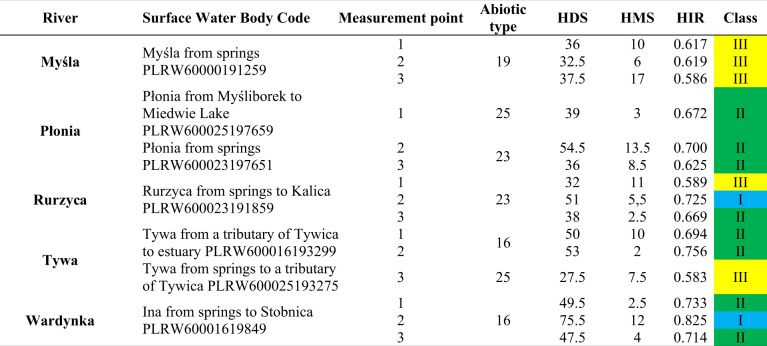
Results of the field assessment for the rivers studied in the Southern Baltic Lake District/Costal Region. The color of the HIR values corresponds to the color from Table 2

### HIR Values for the Watercourse Sections based on the GIS Data Assessment

Through the GIS data assessment of rivers in the SWB (Surface Water Body), the hydromorphological condition class Correction Factor (C_f_) was determined (Tables 6 and 7). The Correction Factor value is influenced by the HDS and HMS values obtained from the GIS data assessment. The highest C_f_ value was recorded at two points (1, 2) on the Tywa River, at 0.62, and it allows the hydromorphological status to be raised by one class for these sections. The high C_f_ value was the result of a high HDS value and a low HMS value in the GIS data assessment. The lowest value (0.14) of the Correction Factor was calculated for all the sections studied on the Zielona River. Such a low value results in a downgrading of hydromorphological class. The low value of HDS_GIS_ (2.33) and high value of HMS_GIS_ (10.0) in the chamber assessment for the Zielona River resulted in the classification of this river to a lower HIR class (IV). In contrast, the highest Hydromorphological Modification Score in GIS data assessment was recorded for the Myśla River (HMS_GIS_ = 16), mainly due to the presence of a large number of hydrotechnical structures and embankments on both sides of the riverbed (>50% of the river length).

**Table 6:** The Correction Factor (C_f_) calculated based on the GIS data assessment for rivers in the Masovian Plain

River	Surface water body code	Measurement point	Abiotic type	HDS_GIS_	HMS_GIS_	C_f_
Czarna Cedron	Czarna Cedron PLRW20001725549	1	17	7.46	12	0.25
		2				
		3				
Kanał Habdziński	Wilanówka PLRW20002625929	1	26	8.32	14.5	0.19
		2				
		3				
Kraska	Jeziorka form springs to Kraska PLRW200017258299	1	17	9.38	13	0.28
		2				
		3				
Molnica	Jeziorka from springs to Kraska PLRW200017258299	1	17	10.38	7	0.51
		2				
		3				
Zielona	Czarna PLRW20001725869	1	17	2.33	10	0.14
		2				
		3

**Table 7:** The Correction Factor (C_f_) calculated based on the GIS data assessment for rivers in the Southern Baltic Lake District/Costal Region

River	Surface water body code	Measurement point	Abiotic type	HDS_GIS_	HMS_GIS_	C_f_
Myśla	Myśla from springs PLRW60000191259	1	19	11.45	16	0.25
		2				
		3				
Płonia	Płonia from Myśliborek to Miedwie Lake PLRW600025197659	1	25	2.87	5	0.33
	Płonia from springs PLRW600023197651	2	23	7.41	6	0.45
		3				
Rurzyca	Rurzyca from springs to Kalica PLRW600023191859	1	23	11.42	13	0.35
		2				
		3				
Tywa	Tywa from a tributary of Tywica to estuary PLRW600016193299	1	16	11.52	5	0.62
		2				
	Tywa from springs to a tributary of Tywica PLRW600025193275	3	25	8.48	10	0.35
Wardynka	Ina from springs to Stobnica PLRW60001619849	1	16	8.45	8	0.41
		2				
		3				
		3				

### The Final HIR Value for the Watercourse Sections, Considering Field Assessment and GIS Data Assessment

Performing a GIS data assessment allowed us to adjust the multimetric HIR value for each section, based on the Correction Factor (Tables 8 and 9). Based on the GIS data assessment, the hydromorphological condition class was downgraded by 1 for 20 sections. According to Table 3, the hydromorphological condition class is downgraded for a Correction Factor ≤0.4. The hydromorphological status class was downgraded for all points except Molnica 1–3, Wardynka 1–3, Tywa 1 and 2, and Płonia 2 and 3. The change concerned four watercourses located in the Middle Vistula water region and four in the Lower Odra and Western Pomerania water region. The downgrading of the watercourses’ hydromorphological class was mainly influenced by the presence of damming structures, river regulating elements, and bridges and embankments detected by the GIS data assessment. At the points Tywa 1 and Tywa 2, the hydromorphological status class was upgraded from II to I. This upgrading resulted from the analysis of the entire river in a given SWB. The GIS data assessment showed a longer natural route for this watercourse than was apparent from the field assessment. In addition, performing a detailed assessment revealed a large proportion of wetlands (10–30%) over the total length of the watercourse, which in the final assessment, resulted in an increase in the degree of naturalness of the watercourse (a change in class from II to I). Compared to the field assessment, where selected sections are analyzed, the GIS data assessment considered more factors that had a significant impact on the final score.

**Table 8:**
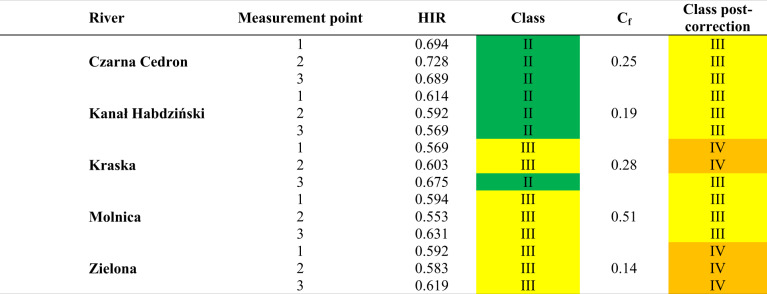
The hydromorphological state class value for rivers in the Masovian Plain after considering the Correction Factor. The color of the class corresponds to the color from Table 2

**Table 9:**
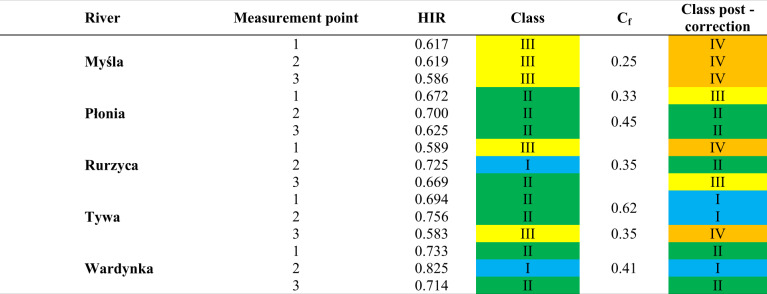
The hydromorphological state class for rivers in the Southern Baltic Lake District/Costal Region after considering the Correction Factor. The color of the class values corresponds to the color from Table 2

### Ward’s Cluster Analysis for Watercourses’ Sections

**Fig. 3:**
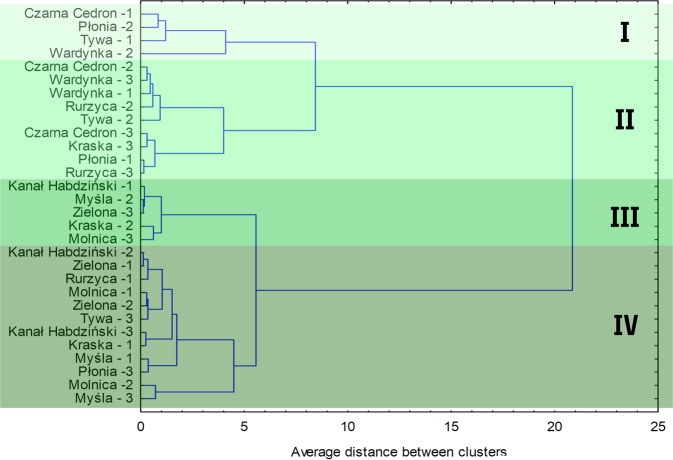
Ward’s cluster analysis of hydromorphological assessment results for the HIR index, based on field survey assessment (the numbers next to the names of the rivers indicate the numbers of the individual study points according to Fig. 1)

The field studies made it possible to classify rivers into three hydromorphological status classes (very good, good and moderate). To better visualize the distribution of the river sections’ hydromorphological status, cluster analysis was carried out using Ward’s method and the individual sections were juxtaposed in the form of a dendrogram (Fig. 3).

Cluster analysis divides the individual sections into 4 main groups. Within each group are sections of watercourses with similar degrees of hydromorphological degradation. The median HDS index in groups I–IV was 52.8, 47.5, 32.5 and 31.3, respectively. HDS values ranged from 49.0–75.5, 38.0–53.0, 27.5–33.0, 27.5–37.5 for groups I, II, III and IV respectively. In turn, the median for the HIR values were 0.694, 0.714, 0.619, 0.588 for groups I–IV, respectively. HIR values ranged from 0.669–0.825, 0.669–0.756, 0.603–0.631, 0.553–0.625. In the case of groups I and IV, the HIR value was also influenced by the high values of the HMS index, which characterizes the degree of modification to the watercourses. In group I, the median HMS index was 12.8 and the range of HMS values in this group is 10.0–15.5, while in group IV the median was 10.0, and the range of HMS values is 7.5–17.5. In groups II and III, the median for the HMS index was 4.0 and 6.0, respectively, and the range of values for this index was 2.0–5.5 and 4.0–6.5, respectively. High HMS values in groups I and IV resulting in lower HIR values despite high HDS index value. As can be seen, more sections belong to groups (III and IV) with a higher degree of hydromorphological degradation (Class III-IV according to the HIR). On the other hand, the sections located in group I and II belong to HIR model hydromorphological degradation classes I and II.

In addition, the features linking the sections of the watercourses in each group were certain morphological parameters, such as the presence of natural morphological elements of the banks, the naturalness and heterogeneity of the valley, and the connection between the river and the valley. The values of these parameters decreased as the group number increased.

### Statistical Analysis of HIR Values and Its Components (HDS and HMS) Considering the Division of the Sections into River Valley Use Types

Table 10 shows the statistical parameters of HDS, HMS and HIR depending on the method used to delineate the river valleys. Note that regardless of the river valley delineation method, the median (Q2) HDS value was highest for seminatural areas (47.5–50.0). The minimum and maximum HDS index values are also higher for seminatural areas than for agricultural areas regardless of how the river valleys are delineated. Similarly for Q1 and Q3, which also take higher values for seminatural than for agricultural areas. On the other hand, the HMS coefficient for the degree of anthropogenic changes in the watercourse and river valley hydromorphology had the highest values for agricultural areas, as confirmed by the data in Table 10. The value of the final HIR depends on both indices (HMS and HDS) which, due to the highest riverbed and river valley diversity and the lowest anthropogenic pressures, was higher for seminatural sites (min. = 0.592–0.694; max. = 0.825; Q1 = 0.672–0.714; Q2 (median) = 0.714–0.728; Q3 = 0.728–0.733) than for agricultural areas (min. = 0.553; max. = 0.756; Q1 = 0.590; Q2 (median) = 0.618–0.619; Q3 = 0.670–0.678).

**Table 10:** Statistical parameters for the river sections: total and by river valley management type determined by two methods

Data set	HDS						HMS						HIR						*n*
	Min	Q1	Q2	Q3	Max	D	Min	Q1	Q2	Q3	Max	D	Min	Q1	Q2	Q3	Max	D	
Total^a^	27.5	31.5	36.0	48.6	75.5	50.0	2.0	4.0	7.8	10.8	17.5	4.0	0.553	0.592	0.622	0.693	0.825	0.694	30
River valley determined by the standard method (according to HIR guidelines)																			
A	27.5	30.8	32.8	39.4	53.0	31.5	2.0	4.0	7.8	10.0	17.5	4.0	0.553	0.590	0.618	0.670	0.756	0.592	24
S	47.5	49.5	50.0	55.5	75.5	–	2.5	4.0	4.0	12.0	15.5	4.0	0.694	0.714	0.728	0.733	0.825	–	5
U	32.0^b^						11.0^b^						0.589^b^						1
River valley determined by novel (author’s) method																			
A	27.5	30.8	34.5	44.5	55.5	29.0	2.0	4.0	7.8	10.4	17.5	4.0	0.553	0.590	0.619	0.678	0.756	0.694	24
S	31.5	39.0	47.5	50.0	75.5	–	3.0	4.0	4.0	10.0	12.0	4.0	0.592	0.672	0.714	0.728	0.825	–	5
U	32.0^b^						11.0^b^						0.589^b^						1

*A* agricultural areas, *S* seminatural areas, *U* urbanized areas, *D* dominant, *n* number of observations, *Q1* quartile 1, *Q2* quartile 2 (median), *Q3* quartile 3

^a^Statistical parameters for all study sections

^b^Basic value for a single measurement point

### Assessing the Impact of Land Use Type and Method for Delineating a River Valley on HDS, HMS and HIR Values

Table 11 shows significant differences in the Mann–Whitney U Test for the river valley determined by the two different methods. From the results in Table 11, it can be concluded that the most significant variance occurs for the HDS parameter when using the standard method of delineating a river valley (*p* = 0.004). This means that a valley’s land use type (agriculture or seminatural) significantly differentiates the HDS value. This parameter, by definition, combines the characteristics of a watercourse and a river valley (Szoszkiewicz et al. 2017) hence the valley’s land use type will have a significant impact on the HDS value. Similarly, there is a significant variance (*p* = 0.002) in the HIR coefficient for the standard method of delineating a river valley. In the case of the novel method for river valley delineation, there is no significance of variance for the HDS (*p* = 0.141) and HIR (*p* = 0.083). In addition to the dominant land use type, the novel method considers the nature of riverbed, its geometry and river valley topography. Hence, the type of land use alone is less significant for the hydromorphological assessment than for the standard method of river valley delineation.

**Table 11:** Results of Mann–Whitney U Test for river valleys delineated by the two methods for HDS, HMS, and HIR coefficients

River valley management (buffer)	HDS	HMS	HIR
River valley determined by the standard method (according to HIR guidelines)			
Agricultural-Seminatural	0.004	0.729	0.002
River valley determined by novel (author’s) method			
Agricultural-Seminatural	0.141	0.544	0.083

Test significance *p* < 0.05

The HMS parameter in both cases (standard and novel method) shows no significant variance against land use type (*p* > 0.05). HMS is responsible for assessing the degree of anthropogenic modification to the watercourse morphology, hence the valley’s land use type will not have much influence on this parameter.

### Correlation between the Components of the HDS and HMS Parameters

Table 12 shows that most of the HDS index components showed weak or low correlation. However, several parameters showed a high correlation. The highest (0.80) was for parameters 3.1 and 3.2, which characterize valley’s land use type and river-valley connectivity. This means that a change in land use around the watercourse will have a significant impact on ensuring the integrity between the riverbed and the valley. Another significant relationship (0.66) relates to parameters 2.1 and 3.1. Parameter 2.1 assesses the structure of bank-top vegetation, which will affect the degree river valley naturalness (3.1) and vice versa. Also associated with parameter 2.1 is parameter 1.8 (correlation - 0.66), which determines the structure of bank vegetation. Both parameters assess the banks in terms of the vegetation present, so it is unsurprising that their values are closely dependent on each other. Other high correlations (0.62–0.63) relate to parameters describing the watercourse itself (1.1) and the morphological elements of the riverbed (1.5) and banks (1.6). Their correlation indicates that changing the morphology of the riverbed and banks will affect the longitudinal variation of the watercourse and the degree of its naturalness.

**Table 12:**
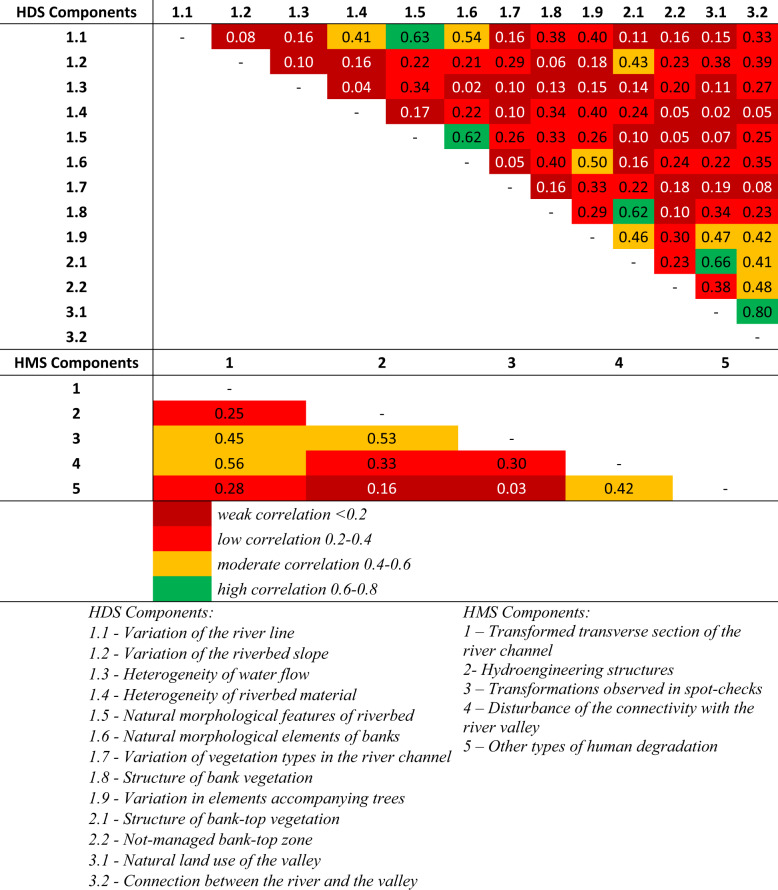
Spearman’s Rank Correlation Coefficient for HDS and HMS components

Analyzing the HMS components (1–5), there is no high correlation. This means that assessing a single component does not allow the degree of hydromorphological modification to be determined. Only the combination of these components into an HMS parameter can give a picture of the watercourse studied. The highest correlations of HMS components were observed for pairs 1–4 (0.56) and 2–3 (0.53). The first pair describes the relationship between the transformation of the transverse section of the riverbed and the disturbance of river-valley connectivity. A moderate correlation indicates some influence of changes to the riverbed (e.g., bank reinforcement), and the free interchange of water between the river and the valley. The second pair is the correlation between the occurrence of hydroengineering structures and transformations in the watercourse profiles. A moderate correlation in this pair may be due to the sporadic occurrence of hydrotengineering structures at the study points. Nevertheless, this correlation is significant in practice and affects the degree to which the watercourse is hydromorphologically degraded.

Table 13 shows the results of Spearman’s rank correlation between the HDS and HMS components. The correlations are similar to those of the components inside each parameter (Table 12). Most of the correlated pairs show a weak and low correlation. This means that analyzing a single component will not provide an accurate hydromorphological assessment of the watercourse. Only the combination of all the components in the HIR parameter will show a realistic picture of hydromorphological degradation. The only pair showing a high correlation coefficient (0.61) is pair 1–1.2. The high correlation is because both components refer to the variation of the riverbed. The same mechanism works for pair 4–3.2 (correlation – 0.60), where each component relates to river-valley connectivity. Pairs 3–1.2 (correlation – 0.59) and 4–1.2 (correlation – 0.54) also relate to the riverbed, hence their moderate correlation. The variation in the riverbed (Component 1.2) will be influenced by hydrotechnical structures (Component 3) and visible transformations in the watercourse profiles (Component 4).

**Table 13:**
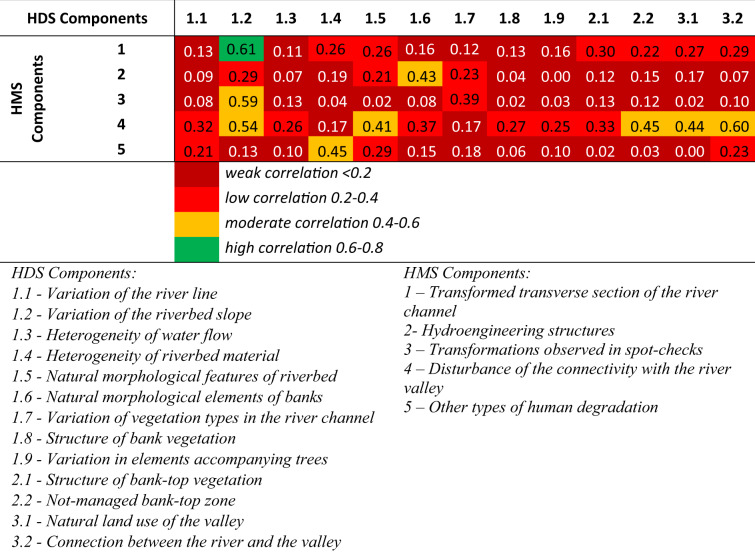
Spearman’s rank correlation between components of HDS and HMS

### Relationship between Abiotic Type of SWB and HDS, HMS and HIR

Table 14 shows the minimum, median and maximum values for HDS, HMS, and HIR according to the abiotic type of the identified SWB. The SWB abiotic type is one of the factors influencing the hydromorphological class of the river. Among the SWB, 6 abiotic types were distinguished: 16 – loess or clayey lowland creek, 17 – sandy lowland creek, 19 – lowland sandy-clay river, 23 – organic creek, 25 – watercourse connecting the lakes, 26 – watercourse in the great river valleys. The highest HDS and HIR values were recorded for streams belonging to loess or clayey lowland creek (16) and organic creek (23). These abiotic types contained mainly seminatural areas, which increase the HDS value. These areas are typical of the grassland and woodland found in river valleys. The sections belonging to watercourses in the great river valleys (26) were slightly differentiated hydromorphologically. For the HMS coefficient, the highest value was recorded for lowland sandy-clay rivers (19), while the lowest anthropogenic pressure was observed in the watercourses connecting the lakes (25).

**Table 14:** Statistical parameters of HDS, HMS and HIR for different abiotic types of SWB

Abiotic type of SWB	*n*	HDS			HMS			HIR		
		Min.	Median	Max.	Min.	Median	Max.	Min.	Median	Max.
16	2	51.5	54.5	57.5	6.0	6.1	6.2	0.725	0.741	0.757
17	3	31.2	32.3	49.5	7.8	8.3	8.5	0.598	0.604	0.704
19	1	35.3^a^			11.0^a^			0.607^a^		
23	2	40.3	41.4	42.5	6.3	8.7	11.0	0.647	0.654	0.661
25	2	27.5	33.3	39.0	3.0	5.3	7.5	0.583	0.628	0.672
26	1	30.5^a^			9.0^a^			0.592^a^		

^a^Basic values for a single SWB

*n* number of delineated SWB

## Discussions

The differences in the rivers’ hydromorphological classes in the areas studied were strongly influenced by their type of management. Those rivers in the Masovian Plain due to the vicinity of the city of Warsaw (in particular the Czarna Cedron and Kanał Habdziński) and large transport routes, are characterized by a lower hydromorphological class (10 points of class III and 5 points of class IV). In contrast, the rivers in the Southern Baltic Lake District/Costal Region are mostly in seminatural or agricultural areas. There is no extensive urban development within several dozen kilometers of the sections surveyed. This is reflected in the quality of these sections and the HIR value (3 points -class I, 5 points - class II, 2 points – class III, and 5 points - class IV). Similar relationships were observed by Pietruczuk et al. (2019). He carried out studies on a lowland river using the HIR model, where the index value was influenced by the type of land use. It should also be added that all the sections of the watercourses surveyed were subject to maintenance works (de-silting, banks mowing, modification of the riverbed) which significantly disturbs their natural character. As Poppe et al. (2016) note, there is a strong correlation between the hydromorphological parameters of watercourses and the extent of their rentauralization.

An important factor affecting hydromorphological conditions is the width of the river valley (Teufl et al. 2013). A wider river valley preserves more consistent features that positively influence morphological conditions, such as longitudinal tree canopies along the watercourse and associated features, or wide floodplains (Pietruczuk et al. 2020). Among the sections studied, it could also be confirmed that larger rivers such as the Czarna Cedron, Płonia, and Wardynka were mostly more morphologically diversified than smaller rivers such as the Myśla, Molnica, Zielona or Kraska. The exceptions are the Tywa and Kanał Habdzinski, of which the Tywa is a small river and falls into HIR class I, while the Kanał Habdzinski, as a small river, has a low HIR value. This means that in addition to the size of the river, the degree of anthropogenic pressures and the diversity of natural morphological elements can also be important. For example, the Wardynka river, in comparison with other watercourses, was subject to occasional maintenance works. Its mountainous character, varied substrate and partially forested catchment allow it to achieve high indices of hydromorphological diversity (Spieczyński et al. 2013). A study conducted a decade ago by Spieczyński et al. (2013) showed that in certain sections the Wardynka river was, hydromorphologically, of moderate ecological status, which did not meet the requirements of the Water Framework Directive. Since then, pro-environmental hydrotechnical works have been carried out to restore river longitudinal connectivity for the migration of fish and other aquatic organisms, which may have affected the morphological diversity of the watercourse.

The way in which the river valley is delineated also affects the hydromorphological assessment of the watercourse by significantly influencing the HDS and HIR parameters. Therefore, this paper attempts to analyze the influence of the river valley land use (determined by two methods) and the individual components of the HIR on the assessment of the hydromorphological condition of watercourses. The standard method of delineating a river valley can be used to simplify analyses, but the novel method is more accurate and reliable. The statistical analyses (Table 10) of HDS, HMS, and HIR given here showed small differences in the values of these indices depending on the method used to delineate a river valley. On the other hand, the Mann–Whitney U Test showed noticeable differences in the variance significance of the HIR components and total HIR with respect to the river valley delineation method. These differences are due to the standard and novel methods’ different degree of accuracy. The standard method assumes that the river valley land use type significantly affects the watercourse hydromorphological assessment indicators. In reality, this is not the only factor which does so. Thus, the standard method is based on a large approximation in delineating a river valley through which there may be greater differences between HDS and HIR values depending on the type of land use. The results of the Mann–Whitney U Test for the novel method of delineating a river valley do not show a strong relationship between the HIR model indices and land use type, as this method consider several other factors that actually shape the hydromorphological conditions of the watercourses. Thus, the novel method is more accurate and better reflects actual valley conditions than the standard method.

In the hydromorphological assessment, the proportion of natural elements within the valleys of the rivers studied and the degree of transformation of the riverbed are highly important (Raczyńska et al. 2012). The statistical analyses performed (Table 10) confirmed that the HDS value is much higher in seminatural than agricultural areas, in contrast to the HMS index. According to Pietruczuk et al. (2019), HDS components have a greater impact on the final HIR than HMS components.

Spearman’s rank correlation analysis of the HDS and HMS individual components revealed that variations in the river line, the natural morphological features of the riverbed, connection between the river and the valley, and the structure of bank and bank-top vegetation have the most significant influence on the hydromorphological assessment. Similar observations appeared in the work of (Raven et al. 2000; Kail et al. 2009; Frankowski 2011; Pietruczuk et al. 2019). The authors of these studies observed that the presence of shrubs, trees and tall herbs improved the morphological conditions of rivers, and the presence of trees and forests increased the number of flow types and the diversity of morphological elements of the banks. Studies by (Jähnig et al. 2010; Lorenz and Feld 2013) confirm that riparian management has a significant impact on the quality of the hydrobiont assemblage and ecological restoration. The connection between the river and the valley plays an important role in hydromorphological assessment. Backwaters and other lentic environments are breeding sites and habitats for endangered species, and thus they determine the ecological status of the river and adjacent areas and the biodiversity of the aquatic ecosystem (Jelonek 2002).

The statistics describing the relationship between river abiotic type and HIR value showed that the highest HIR values were observed for abiotic type 16 and the lowest for abiotic types 19 and 26. Previous studies by Pietruczuk et al. (2020) have shown that as the width of riverbed increases, the amount of channel and bank reinforcement increases, i.e., greater hydromorphological transformation is observed on larger rivers. Hydromorphological transformations negatively affect the HIR value, so the HIR value is lower in larger rivers (type 26). In Poland, lowland areas with sandy-clay soils (type 19) are mainly agricultural and partially urbanized areas; hence, rivers flowing through these areas often require regulation which affects the high HMS value and lower HIR value. On the other hand, around lakes (type 25), there is not much anthropogenic pressure due to the frequent occurrence of protected habitats for flora and fauna. Watercourses classified as abiotic type 16 are characterized by a small catchment area, a winding or meandering course, a seminatural character and high bottom erosion where the riverbank cuts deeply into the valley bottom. They are rarely found outside forested areas (Błachuta et al. 2010). The above characteristics influence the higher HIR value of abiotic type 16 compared to the others.

## Conclusions

The study of watercourses using the HIR model in two different regions of Poland showed significant differentiation in their hydromorphological status. The assessment of this status was mostly influenced by the share of natural elements within the river valley of the river studied, the degree of transformation of the riverbed and the management of the river valley. In the manuscript, a novel method for delineating river valleys has been proposed. The novel method is more accurate and reliable than standard method because it is based on a detailed map analysis. The delineation of river valleys using the novel method can contribute to more reliable HIR model results. The hydromorphological condition of the watercourses was evaluated using a one-time field assessment. To accurately assess progressive hydromorphological processes and determine the degree of change, annual surveys would need to be conducted for at least a dozen years using the same model. It is particularly important to consider climate change, which in addition to anthropogenic pressures is significantly affecting and modifying the hydromorphology of watercourses (Raven et al. 1998). The results presented in this paper confirmed the reliability and versatility of the HIR model for lowland rivers. Conducting hydromorphological studies has a special impact on water management. It allows us to assess whether watercourses require renaturalization, which results in the improvement of water retention, the increase of hydromorphological diversity and the restoration of the continuity of watercourses.
